# Supplementary material for: Model-based estimation of left ventricular pressure and myocardial work in aortic stenosis
Source: PLoS One. 2020 Mar 3;15(3):e0229609. doi: 10.1371/journal.pone.0229609 (PMC7053724; doi:10.1371/journal.pone.0229609)
Supplement: S1 File — (PDF) [file pone.0229609.s003.pdf]

Table 1. Parameters Par_{art} identified for patients 1 to 4

	Pat 1	Pat 2	Pat 3	Pat 4
R_{sys}(mmHg.s/ml)	0.76 ± 0.07	0.83 ± 0.07	0.87 ± 0.05	0.83 ± 0.07
E_{ao}(mmHg/ml)	3.66 ± 0.6	3.5 ± 0.75	2.64 ± 0.63	3.15 ± 0.58
E_{sa}(mmHg/ml)	0.88 ± 0.23	0.81 ± 0.19	0.84 ± 0.23	0.82 ± 0.22
E_{sv}(mmHg/ml)	0.017 ± 0.004	0.016 ± 0.004	0.02 ± 0.004	0.02 ± 0.004
E_{vc}(mmHg/ml)	0.012 ± 0.0015	0.012 ± 0.002	0.01 ± 0.002	0.01 ± 0.002
$V_{d,ao}$(ml)	211.6 ± 39	219.9 ± 40.98	216.7 ± 48.4	221.2 ± 47.3
$V_{d,sa}$(ml)	549.6 ± 133.9	509.1 ± 137.5	565.01 ± 130.8	550.6 ± 134.9
$V_{d,sv}$(ml)	2131.7 ± 367	2317.9 ± 380	2298.3 ± 371.3	2224.5 ± 394
$V_{d,vc}$(ml)	1893.6 ± 321	2025.7 ± 292	1844.5 ± 334	1791.5 ± 339
R_{ao}(mmHg.s/ml)	0.34 ± 0.05	0.33 ± 0.06	0.32 ± 0.07	0.29 ± 0.06
R_{vc}(mmHg.s/ml)	0.17 ± 0.04	0.16 ± 0.04	0.15 ± 0.04	0.18 ± 0.05

Table 2. Parameters Par_{art} identified for patients 5 to 8

	Pat 5	Pat 6	Pat 7	Pat 8
R_{sys}(mmHg.s/ml)	0.8 ± 0.08	0.85 ± 0.07	0.87 ± 0.05	0.66 ± 0.07
E_{ao}(mmHg/ml)	2.86 ± 0.82	2.67 ± 0.76	2.79 ± 0.62	3.77 ± 0.48
E_{sa}(mmHg/ml)	0.79 ± 0.25	0.85 ± 0.27	0.82 ±0.23	0.91 ± 0.22
E_{sv}(mmHg/ml)	0.015 ± 0.003	0.016 ± 0.004	0.016 ± 0.004	0.015 ± 0.003
E_{vc}(mmHg/ml)	0.01 ± 0.002	0.011 ± 0.002	0.012 ± 0.002	0.011 ± 0.0016
$V_{d,ao}$(ml)	202.7 ± 47.9	217.9 ± 43.9	209.4 ± 55.01	201.3 ± 51.3
$V_{d,sa}$(ml)	526.7 ± 119.1	544.4 ± 137.1	518.5 ± 143.3	519.5 ± 109.2
$V_{d,sv}$(ml)	1995.6 ± 452	2267.8 ± 440	2422.3 ± 351	2124.1 ± 317
$V_{d,vc}$(ml)	1765.1 ± 361	1811.6 ± 374	1963.2 ±283	1789.3 ± 360
R_{ao}(mmHg.s/ml)	0.29 ± 0.06	0.32 ± 0.07	0.31 ± 0.06	0.33 ± 0.035
R_{vc}(mmHg.s/ml)	0.19 ± 0.04	0.17 ± 0.05	0.17 ± 0.04	0.17 ± 0.042

Table 3. Parameters Par_{art} identified for patients 9 to 12

	Pat 9	Pat 10	Pat 11	Pat 12
R_{sys}(mmHg.s/ml)	0.71 ± 0.09	0.78 ± 0.07	0.86 ± 0.07	0.87 ± 0.06
E_{ao}(mmHg/ml)	3.22 ± 0.67	3.13 ± 0.59	1.94 ± 0.44	2.16 ± 0.71
E_{sa}(mmHg/ml)	0.88 ± 0.18	0.86 ± 0.20	0.74 ± 0.21	0.80 ± 0.24
E_{sv}(mmHg/ml)	0.015 ± 0.003	0.02 ± 0.004	0.016 ± 0.004	0.016 ± 0.004
E_{vc}(mmHg/ml)	0.01 ± 0.003	0.01 ± 0.002	0.012 ± 0.002	0.011 ± 0.002
$V_{d,ao}$(ml)	202.8 ± 44	211.8 ± 46.5	213.4 ± 50.1	185.3 ± 56.03
$V_{d,sa}$(ml)	543.1 ± 117.4	533.9 ± 120.9	529.9 ± 155.1	584.4 ± 129.1
$V_{d,sv}$(ml)	2001.8 ± 390	2156.8 ± 357	2259.6 ± 425	2327.7 ± 421
$V_{d,vc}$(ml)	1533.2 ± 382	1789.6 ± 364	1828.4 ± 333	1904.7 ± 314
R_{ao}(mmHg.s/ml)	0.29 ± 0.06	0.30 ± 0.05	0.23 ± 0.07	0.26 ± 0.08
R_{vc}(mmHg.s/ml)	0.19 ± 0.04	0.18 ± 0.05	0.19 ± 0.05	0.17 ± 0.04

Table 4. Parameters Par_{LV} identified for patients 1 to 6

	Pat 1	Pat 2	Pat 3	Pat 4	Pat 5	Pat 6
E_{lv}(mmHg/ml)	3.4738	2.7828	3.0753	1.8953	3.2923	2.5773
λ_{lv}(1/ml)	0.0149	0.0146	0.0189	0.0105	0.0137	0.0127
$P_{0,lv}$(mmHg)	1.4422	1.3184	0.9674	1.0494	1.5145	0.9559
α_1	0.3077	0.3517	0.3523	0.5141	0.3283	0.3238
α_2	0.4107	0.42	0.3139	0.3257	0.37	0.3646
n_1	1.2798	1.2381	1.3637	1.3934	1.1412	1.2695
n_2	24.2611	21.6158	24.9414	15.6806	13.8802	16.7110

Table 5. Parameters Par_{LV} identified for patients 7 to 12

	Pat 7	Pat 8	Pat 9	Pat 10	Pat 11	Pat 12
E_{lv}(mmHg/ml)	3.0198	3.2276	3.3745	2.4147	3.1468	3.6446
λ_{lv}(1/ml)	0.0172	0.0133	0.0093	0.0188	0.0152	0.0154
$P_{0,lv}$(mmHg)	1.4185	1.7418	1.13	1.9088	0.7194	1.5269
α_1	0.2420	0.4712	0.5210	0.3781	0.3388	0.3359
α_2	0.3409	0.4416	0.3898	0.4268	0.3919	0.4237
n_1	1.5742	1.5367	1.1278	1.3258	1.8088	1.1276
n_2	19.2338	12.0314	20.9134	12.7301	16.6476	24.4148

Table 6. Model parameters

ρ (g/cm^3)	1.06
$K_{vc,aortic}$ (1/Pa.s)	0.15
$K_{vo,aortic}$ (1/Pa.s)	0.12
$l_{eff,aortic}$ (cm)	2.2
$K_{vc,tricuspid}$ (1/Pa.s)	0.4
$K_{vo,tricuspid}$ (1/Pa.s)	0.3
$l_{eff,tricuspid}$ (cm)	2
$K_{vc,mitral}$ (1/Pa.s)	0.4
$K_{vo,mitral}$ (1/Pa.s)	0.3
$l_{eff,mitral}$ (cm)	1.25
$K_{vc,pulmonary}$ (1/Pa.s)	0.2
$K_{vo,pulmonary}$ (1/Pa.s)	0.2
$l_{eff,pulmonary}$ (cm)	1.9
A_{eff,max_mitral} (cm^2)	5
$A_{eff,max_tricuspid}$ (cm^2)	6
$A_{eff,max_pulmonary}$ (cm^2)	2.8
$V_{d,lv}$ (ml)	10
$V_{0,lv}$ (ml)	10
E_{rv}(mmHg/ml)	0.6526
$V_{d,rv}$ (ml)	10
$V_{0,rv}$ (ml)	10
λ_{rv}(1/ml)	0.013
$P_{0,rv}$ (mmHg)	1.2001
E_{pa}(mmHg/ml)	0.3375
$V_{d,pa}$ (ml)	160
E_{pv}(mmHg/ml)	0.0062
$V_{d,pv}$ (ml)	200
R_{pul}(mmHg.s/ml)	0.1425
C_{la} (s)	0.32
B_{la} (1/s^2)	84.375
$E_{la,max}$(mmHg/ml)	1.6
$E_{la,min}$(mmHg/ml)	0.1
$V_{d,la}$ (ml)	3
R_{la}(mmHg.s/ml)	0.01
$E_{ra,max}$(mmHg/ml)	1.6
$E_{ra,min}$(mmHg/ml)	0.1
$V_{d,ra}$ (ml)	3
R_{ra}(mmHg.s/ml)	0.01
