# Supplementary material for: Model-based estimation of left ventricular pressure and myocardial work in aortic stenosis
Source: PLoS One. 2020 Mar 3;15(3):e0229609. doi: 10.1371/journal.pone.0229609 (PMC7053724; doi:10.1371/journal.pone.0229609)
Supplement: Attachment — Submitted filename: Response_to_Reviewers.pdf [file pone.0229609.s004.pdf]

Reviewer #1:

In this paper, Owashi et al. performed a model-based estimation of LV pressure and myocardial work that can be applied in the context of patients with aortic stenosis (AS). They validated their model in a cohort of 12 patients with AS (11 severe and 1 moderate AS). Authors reported model errors around 12% for the estimation of LV pressure.

I have few comments for the authors regarding the present manuscript.

1 – Authors reported a good correlation between estimated and measured pressure (end of page 10). However, even if the correlation coefficient was relatively high (>0.90), the intercept of the slope pointed out an underestimation ranged between 8 to 9 mmHg. Similar findings are identified in the second step of the analysis. Was this difference between model-based estimation and measured pressure clinically relevant? What was the clinical significance of this "systematic" underestimation? How can we ascertain the low clinical impact of this kind of LV pressure underestimation, especially in a context of AS?

We thank reviewer 1 for this interesting question. As shown in previous work of our team (Hubert 2018), although LV pressure is imprecise, the estimation of LV work could be accurate. In fact, even errors between model-based and measured pressures are around 12%, mean bias in BA analysis were -140 mmHg.% and -12 mmHg.% respectively for GCW and GWW, which correspond to relative bias equal to 3.47 % and 2.93 % with respect to maximum GCW and GWW. Mean bias for GWE is equal to 0. The consistency of LV work estimation could be explained by: i) the temporal integration, which induces a smoothing of the difference between measured and estimated works and ii) relative precision of the estimation of the pressure between AVO and AVC. Although the estimation of the LV pressure is imperfect, the non-invasive estimation of global myocardial work indices obtained from modelling approach strongly correlates with invasive measurements and we believe that the proposed estimation of LV myocardial work is clinically relevant. Discussion section was modified in order to better consider these aspects.

Hubert A et al. Estimation of myocardial work from pressure-strain loops analysis: an experimental evaluation. Eur Heart J Cardiovasc Imaging. 2018;doi:doi: 10.1093/ehjci/jey024.

2 – Could you validate your estimation of myocardial work based on clinical gold standard currently used in clinic?

The gold standard consists in measuring invasively left ventricular myocardial deformation by

sonomicrometry simultaneously with left ventricular pressure by micromanometer. In our study, LV pressure was measured because of clinically utility but it would be not ethical to realize sonomicrometry in patients with aortic stenosis for an observational study. Unfortunately, this gold standard is impossible in human, only in animals, as Urheim et al (2005) did for normal LV.

Urheim S et al. Regional myocardial work by strain Doppler echocardiography and LV pressure: a new method for quantifying myocardial function. American Journal of Physiology - Heart and Circulatory Physiology. 2005;288:H2375–H2380.

3 – What is the additive value of the estimation of myocardial pressure or work as compared to echo based data including AVA and LV function parameters? A validation of the clinical impact of this approach will considerably strengthen the paper.

Timing and indications for surgical intervention in patients with severe asymptomatic AS without LV dysfunction remains highly controversial. In fact, surgery is currently not recommended despite in the increased risk of cardiovascular mortality in this population (Kang DH et al., 2019). Global longitudinal strain appears to have a better significance than left ventricular ejection fraction to predict events in this population (Magne J et al., 2019). But it's well known that GLS is afterload dependant so our hypothesis is that myocardial work, taking in count LV afterload, will better stratify pre-operative and post-operative risk in this population. In fact, because afterload data are included in the calculation of myocardial work in the form of LV pressure, the assessment of myocardial work might represent a more robust parameter with respect to the assessment of LV strain or other strain-derived parameters.

Discussion was modified in order the better describe the clinical objectives of this approach.

Kang DH et al. Early surgery or conservative care for asymptomatic aortic stenosis. NEJM 2019, DOI: 10.1056/NEJMoa1912846

Magne J et al. Distribution and Prognostic Significance of Left Ventricular Global Longitudinal Strain in Asymptomatic Significant Aortic Stenosis: An Individual Participant Data Meta-Analysis. JACC Cardiovasc Imaging. 2019;12:84–92

Galli, E, Leclercq, C, Fournet, M, Hubert, A, Bernard, A, Smiseth, OA, Mabo, P, Samset, E, Hernandez, A, Donal, E (2018). Value of Myocardial Work Estimation in the Prediction of Response to Cardiac Resynchronization Therapy. J Am Soc Echocardiogr, 31, 2:220-230.

4 – What is the efficacy of the model in patients with a more advance stage of the LV dysfunction?

For this first approach of the model, we decided to have a homogeneous population so aortic

stenosis with normal ejection fraction. But this question is very interesting and should be adressed in further studies.

5 – Authors provided echo data for each patient in a supplemental table. The LVEDV/LVESV should be carefully reviewed by the authors. How did you calculate LVEF? SV and strain data could be of interest.

Indeed, there was an inversion between LVEDV and LVESV in this table. Left ventricular ejection fraction was calculated by manual Simpson's biplane, realized by a certified cardiologist and followed current recommendations (Lang RM et al., 2015). The data table (data_PlosOne.xls) was modified in the supporting information.

Lang RM et al. Recommendations for Cardiac Chamber Quantification by Echocardiography in Adults: An Update from the American Society of Echocardiography and the European Association of Cardiovascular Imaging. Eur Heart J Cardiovasc Imaging. 2015;16:233–271

Reviewer #2:

This paper describes a model-based approach for estimating left ventricular constructive and wasted work, and work efficiency, in patients with aortic stenosis. The model is very well suited to the question and the results are quite impressive. The main limitation is the small sample size of only 12 patients; it is therefore difficult to tell how robust the results are.

We would like to thank the reviewer and we have modified the manuscript in order to improve the article.

Concerning the small sample size, the low number of patients is due to the difficulty of measuring LV pressure in aortic stenosis because current guidelines discourage catheterization measurement techniques. In our study, LV pressure was measured because of clinically utility and all patients were informed.

1. Line 122. The equation for calculating volume is missing a reference volume. The integral of flow can only be used to calculate a volume change, not an absolute volume.

The reviewer is right. The equation describes de flow change: $\Delta V(t) = \int (Q_{in} - Q_{out}) dt$. Section 1.2.3 was corrected.

2. I assume Section 1.3 relates to the clinical data. Please make this clear at the start of this section, as it follows the model description.

Section 1.3 was modified in order to improve the description of work indices calculated from clinical and simulated data: Segmental myocardial work, as proposed by Russell et al (Russell 2013), could be calculated from 1) the clinical strain signals, deduced from TTE, and 2) the LV pressure obtained invasively by catheterization P_{LV}^{exp} or the patient-specific pressure P_{LV}^{model} from the model-based approach.

Russell K, Eriksen M, Aaberge L, Wilhelmsen N, Skulstad H, Gjesdal O, et al. Assessment of wasted myocardial work: a novel method to quantify energy loss due to uncoordinated left ventricular contractions. Am J Physiol Heart Circ Physiol. 2013;305:H996–H1003,.

3. How were the S and IVR periods of the cardiac cycle defined?

The definition of S and IVR period were integrated in the legend of figure 2. S phase is defined by the time interval spanning from mitral valve closure (MVC) to aortic valve closure (AVC), whereas the IVR phase is defined between aortic valve closure and mitral valve opening (MVO).

4. Line 170-171. "For each parameter Xj, a number r of elementary effects are calculated to estimate the mean (μ∗) and standard deviation (σ) of the effects." I don't quite follow this statement. Do the authors mean that a range of Xj values are tested and then the average effect (and SD) is taken as a measure of sensitivity? If so, how was the range of Xj values determined? And was Xj adjusted up and down? If so taking the mean would be inappropriate.

The method consists in the generation of several random trajectories through the parameter space; each trajectory being associated with an estimation of the Elementary Effects EE^*_j of a parameter X_j on output Y :

$$EE_j^* = \left|\frac{Y(X_1, \dots, X_j, \dots) - Y(X_1, \dots, X_j + \Delta, \dots)}{\Delta}\right|$$

where Δ is a predefined variation. For each X_j , the mean μ^*_j and standard deviation σ_j of r elementary effects (EE_j) are calculated. A large value of μ^*_j indicates a significant effect of X_i on Y , whereas a large σ_j value is related to either non-linear or strongly interacting variables. In order

to establish a global rank of importance among parameters, we calculated the Euclidean distance D_j in the $\mu^* - \sigma$ plane, from the origin to each (μ^*_j , σ_j) point. For each parameter X_j, the range of possible values was defined as +/- 30% of the initial value (supplementary materials).

Section 1.4.1 was modified.

5. Figure 4 specifies a) and b), but those labels are not present. Use of (top) and (bottom) is sufficient.

Legend of figure 4 was corrected.

6. In Section 2.3 it is unclear why R^2 values are reported, as these are unreliable measures of agreement. E.g. High R^2 can be obtained even when there is poor agreement, as in the two bottom left panels of Figure 7. e% values are better. I may have missed it, but what is beta? It would be helpful to remind readers of the error measures in the Figure legend.

The goodness of fit was evaluated by performing a linear regression using all the data points from the pressure waveforms, and the coefficient of determination (R^2), as well as the slope (β), were determined. The R^2 and β values were removed from figure 6 and 7 and only e% values appear in these figures. Results are presented in sections 2.3.1 and 2.3.2.

7. In Section 2.4, it may be helpful to also explain the biases in relative terms, as I (and perhaps other readers) don't have a feel for whether -140 mmHg.% is a big or small number.

In BA analysis, mean bias were -140 mmHg.% and -12 mmHg.% respectively for GCW and GWW, which correspond to relative bias equal to 3.47 % and 2.93 % with respect to maximum GCW and GWW values. Section 2.4 was modified to explain the biases in relative terms.

8. Line 272. "For GWE, all patients are within the 95% limits of agreement whereas, for GCW and GWW, one patient is outside". This statement is tautological, as by definition the 95% limits of agreement will always contain all or almost all of the data for a sample of this size. The authors may want to consider whether the one data point could be defined as an outlier, but this would be difficult to judge from such a small sample.

The reviewer is right. This sentence was removed from the manuscript.

9. Line 274-276. Please check whether this text does indeed refer to the third patient in the first

column of Figs 6 and 7, as the timing of the peaks for this case seem to be pretty well aligned, more so than many of the others.

In fact, there was a mistake in this sentence, as it is the third patient in the first row of Figs 6 and 7. Section 2.4 was corrected.

10. Line 334. “we can suppose that myocardial works will be robust complementary indices independ[e]nt of afterload condition.” This may be so, but I would suggest the authors discuss what needs to be done to establish evidence around this question.

Concerning the influence of loading conditions, it is recognized that the estimation of myocardial work provides an automatic analysis of myocardial performance that is independent of LV afterload (Galli, 2018). Because afterload data are included in the calculation of myocardial work in the form of LV pressure, the assessment of myocardial work might represent a more robust parameter with respect to the assessment of LV strain or other strain-derived parameters. The discussion section was modified.

Galli, E, Leclercq, C, Fournet, M, Hubert, A, Bernard, A, Smiseth, OA, Mabo, P, Samset, E, Hernandez, A, Donal, E (2018). Value of Myocardial Work Estimation in the Prediction of Response to Cardiac Resynchronization Therapy. J Am Soc Echocardiogr, 31, 2:220-230.

11. Line 342. Replace “it is necessary to precise that” with “it is necessary to recognise that”.

The sentence was modified in the manuscript.

12. Line 347-350. It is surprising that filling pressure has no impact on myocardial work. Preload should have a large impact on developed pressure and cardiac output, hence I would have expected a significant sensitivity to this. Can the authors please clarify and explain this?

We agree with the reviewer. In fact, this sentence is ambiguous and the discussion section was modified. Despite the estimation of filling pressure remains imperfect, myocardial work is considered in the period from MVC to MVO, so inaccuracies before mitral valve closure and after mitral valve opening has no impact on the results.

13. Check references. A number of references have question marks (?) in various places.

The references section was corrected.

Minor:

14. Line 255. change “was equal 11.9% to” to “was equal to 11.9%”

15. Line 298. Change to “the most influential”

16. Line 326. Replace “indice” with “index”

17. Throughout the manuscript, replace “works” with “work”. E.g. “constructive and wasted myocardial work” is correct English.

We thank the reviewer for these suggestions. We have integrated all these corrections in the manuscript.
