# Supplementary material for: Model-based estimation of left ventricular pressure and myocardial work in aortic stenosis
Source: PLoS One. 2020 Mar 3;15(3):e0229609. doi: 10.1371/journal.pone.0229609 (PMC7053724; doi:10.1371/journal.pone.0229609)
Supplement: Attachment — Submitted filename: Response_to_Reviewers.pdf [file pone.0229609.s005.pdf]

Reviewer #1:

Almost all the comments have been addressed. The remaining point that authors should, at least, acknowledge is lack of validation of the additive predictive value of the estimation of myocardial work (comment #3). Authors provided in their response and the revised manuscript, several hypotheses that could support the usefulness of the estimation of myocardial work, but this should be validate in a cohort of AS patients. Do you have access to an external cohort of AS patients in which myocardial work could be estimated and then in which the additive predicting value could be tested? This would significantly re-inforce the message of the paper. At least, this should clearly be acknowledged by the authors.

We thank reviewer 1 for his comments. The objectives of this paper are 1) to propose a novel tool to estimate non-invasively myocardial work and 2) to compare model-based and invasive indices. This article does not claim to prove the additive predicting value of myocardial work estimation in aortic stenosis. The reviewer is right because this would significantly re-inforce the message of the paper. Unfortunately, we don't have currently access to any database of AS patients that incudes both echocardiographic strains and invasive ventricular pressures. We are working on the definition of a prospective study for the validation of proposed indices on AS patients and this article is the first step of our approach. We also believe that it is important to clarify the objectives of the paper and to acknowledge that our article does not claim to validate the estimation of myocardial work in a cohort of AS patients. We add several sentences in the introduction and the discussion in order to clarify these aspects.
